# Effective Treatment Approaches for Eating Disorders in Children and Adolescents: A Review Article

**DOI:** 10.7759/cureus.74003

**Published:** 2024-11-19

**Authors:** Yara Alharbi, Fatema Saleh, Khaled A Shahat

**Affiliations:** 1 College of Medicine, Taibah University, Madinah, SAU; 2 Department of Pediatrics, College of Medicine, Taibah University, Madinah, SAU; 3 Pediatrics, Al Rayan National College of Medicine, Madinah, SAU

**Keywords:** anorexia nervosa, avoidant-restrictive food intake disorder, binge eating disorder, bulimia nervosa, eating disorder, pharmacotherapy, psychotherapy

## Abstract

Eating disorders are serious psychiatric illnesses marked by disordered behaviors toward food and eating due to dissatisfactory body shape and weight, which impact the physical and psychological growth of children and adolescents. This review aims to recognize the effectiveness of psychotherapy and pharmacotherapy in treating eating disorders. The most common type of eating disorder is anorexia nervosa characterized by severe restriction of energy intake and an intense fear of gaining weight. Bulimia nervosa is characterized by episodes of binging and purging followed by compensatory behaviors. Binge eating disorder is characterized by binging without compensatory behaviors. Avoidant-restrictive food intake disorder is characterized by a lack of interest in eating unrelated to weight and shape concerns. Depending on the severity of the condition, patients could be treated as an inpatient, partial hospitalization, or outpatient treatment using different psychotherapies, including family-based therapy, psychodynamic individual treatment, cognitive behavioral therapy, adolescent-focused therapy, interpersonal psychotherapy or pharmacotherapy, or a combination of both. Studies in children and adolescents show that family-based therapy is considered the first-line treatment for anorexia nervosa, and the second evidence-based approach is adolescent-focused therapy. As for bulimia nervosa, both family-based treatment and cognitive behavioral therapy are almost equally effective. In binge eating disorder, cognitive behavioral therapy and interpersonal psychotherapy are the most beneficial psychotherapies. For avoidant-restrictive food intake disorder, psychotherapy or hospitalization could be considered. Concerning pharmacotherapy, none of the medications have been approved by the US Food and Drug Administration for children and adolescents.

## Introduction and background

Eating disorders are serious psychiatric illnesses marked by disordered behaviors toward food and eating due to dissatisfactory body shape and weight [1]. These affect the physical and psychological growth of children and adolescents [2]. They are potentially life-threatening disorders with a high rate of mortality and morbidity due to cardiovascular complications of malnutrition, which can lead to cardiac arrest. At the same time, depression is frequently comorbid with eating disorders, and suicide is unfortunately common in the patient population [3,4].

Because of media exposure, Western influences, and obesity, the frequency of eating disorders has risen dramatically in the last two decades around the world, affecting people from all socioeconomic, cultural, and ethnic groups. Despite the significant prevalence of disordered eating attitudes, there has been a scarcity of research in Arab nations, including Saudi Arabia. However, a few research projects have been undertaken in Arar, Jeddah, Riyadh, and Hail. In a study from Arar, Saudi Arabia, 314 female teenagers (aged 15 to 19 years) were chosen from four schools. The participants were asked to complete a pre-tested questionnaire regarding their socioeconomic status, eating habits, and the Eating Attitude Test 26 (EAT 26). Additionally, the study assessed their height and weight and calculated their body mass index (BMI). Overall, 25.47% of subjects had disrupted eating habits (EAT-26 >20) [5].

In Jeddah, Saudi Arabia, another study conducted in three secondary schools utilized a cross-sectional design. A total of 425 female adolescents were included in the study. The results revealed that 32.9% of the study population received a score of 20 or above on the Arabic version of the self-report EAT-26 questionnaire. This finding implied that they were in danger of developing eating disorders. The mean of the participants’ BMI and their Eating Attitude whole scale and three subscale scores were found to have a significant association (dieting, bulimia, and oral control). Furthermore, a negative significant association was discovered between the average age of the participants and their total Eating Attitude scale scores [6].

In another study, female students in Riyadh, Saudi Arabia, were selected using a stratified cluster selection method. The EAT-26 was completed, and the subjects’ heights and weights were measured. The total sample size was 1,271 students, with 67.8% of them completing the questionnaire to the point where a factor analysis could be performed. The result showed that the positive rate for the EAT-26 was 24.6%, with more Eating Attitudes test-positive cases having had more interaction with the West and being able to communicate in a Western language [7].

In a study from Hail, a representative sample of Saudi teenagers and young adults was recruited. A modified version of the EAT-26 was used in a cross-sectional survey of adolescents using face-to-face interviews. A national sample of 100 young individuals aged 18 to 25 years old was taken. The overall prevalence of eating disorders was estimated to be 2% and 24% for males and females, respectively [8].

Non-clinical research has also indicated that 36.7%, 33%, and 21% of people in the United Arab Emirates (UAE), Jordan, and Sudan, respectively, have aberrant eating attitudes and habits. Another study from UAE unveiled that 23.4% and 66% of adolescent girls were engaged in negative eating attitudes and desired to be thin, respectively. Furthermore, the risk of disordered eating attitudes among teenagers was found to be twice as high among girls as in boys, and the risk of disturbed eating attitudes among obese adolescents was two to three times greater than that of non-obese adolescents in both genders [5].

Eating disorders come in a variety of forms, the most common of these is anorexia nervosa (AN), which is defined as restricting food intake in relation to energy requirements, resulting in severe weight loss and a fear of gaining weight. Patients with bulimia nervosa (BN) have bouts of binge eating that are followed by recurring compensatory measures such as vomiting, fasting, laxatives, or exercise to avoid weight gain. Weight and shape have a significant impact on self-evaluation, and these episodes occur at least once every week over 12 weeks. Binge eating disorder (BED) is characterized by recurrent binge eating (at least once a week for 12 weeks) with no compensatory activity. Avoidant-restrictive food intake disorder (ARFID) is a new diagnosis in the Diagnostic and Statistical Manual of Mental Disorders, fifth edition (DSM-5) that is characterized by restrictive eating unrelated to body image or weight and shape concerns. Instead, ARFID is a relatively new diagnosis defined by the failure to achieve basic daily nutritional requirements due to a lack of interest in eating and avoidance of food with specific sensory features (color, texture, smell, taste, or fear of choking) [9].

Various biological, psychological, and societal factors play a role in the development of eating disorders. These factors may interact differently among different individuals, resulting in extremely varied thoughts, experiences, and symptoms in two individuals with the same eating disorder [10]. The diagnosis of eating disorders is based on signs, symptoms, and eating habits, and when eating disorders are suspected, doctors perform physical examinations and laboratory tests to rule out other medical causes. The psychological evaluation by the DSM-5 criteria and other assessments is a powerful tool for diagnosis. There is still a lot to learn about the origins, development, and successful management of eating disorders. Feeding disorders are frequently treated with a combination of psychotherapy and pharmacotherapy [11]. Suitable treatment choices depend on the improvement of weight and distorted thinking.

## Review

Treatment for anorexia nervosa

*Inpatient Treatment*

The primary goal of hospital-based stabilization is nutritional restoration. It is critical to strike a balance between two competing goals, namely, rapid weight gain and avoidance of refeeding syndrome. Refeeding syndrome refers to the metabolic and clinical changes that occasionally occur when a malnourished patient is aggressively nutritionally rehabilitated. Typically, it takes between four to six weeks for an average child or adolescent to accomplish these goals. However, in reality, pressure to discharge the patient increases after several days due to third-party payment agents and utilization review committees, among other reasons, with the length of stay limited to one or two weeks at most [12,13].

Hospitalization should be considered if any one or more of the following clinical circumstances appear: extreme malnutrition, in which the child’s weight is less than three-quarters of his or her optimal body weight, especially if weight loss is rapid, besides those low chances of reversing weight loss on an outpatient basis, due to factors such as degree of determined food avoidance and previous clinic-based therapy failures, as well as health problems such as dehydration, electrolyte imbalances, abnormal heart rhythm, slow heart rate, low blood pressure, extreme drop in body temperature, neurological complications, and reduced pulse rate. Finally, comorbid mental illnesses or suicidal thoughts may interfere with outpatient therapy.

The treatment of children and adolescents in hospitals is still not clear. According to multicenter research collaboration in the United States, those who were hospitalized in a national cohort of low-weight nine- to 21-year-olds with restrictive eating disorders had a higher likelihood of being at 90% of the median BMI at the one-year follow-up. However, a randomized controlled trial (RCT) from the United Kingdom among adolescents with AN found no benefits of inpatient care over outpatient care; this study was limited by poor adherence to the assigned treatment. An RCT conducted in Germany in 2014 discovered that inpatient adolescents who were discharged earlier to outpatient treatment performed as well as those who were discharged later. Similarly, an RCT conducted in Australia in 2015 found that adolescents who were discharged to family-based therapy (FBT) as soon as they were medically stable performed at least as well as adolescents who remained inpatients until they reached 90% of their treatment goal weight [14-16].

Clinical experience has shown that refeeding hospitalized patients with anorexia is often easier than refeeding in clinic-based care owing to the greater degree of regulation that can be achieved in the hospital. During the hospitalization, families that observe hospital professionals implement an organized nutrition plan are more equipped to sustain it upon release compared to families who construct an organized eating program under the supervision of an outpatient therapist. In the literature, there are no controlled, random-assignment trials examining the effectiveness of the type of comprehensive inpatient therapy described here. Two important studies that examined the use of specialized inpatient programs for AN in comparison to outpatient therapies found no evidence of a difference in benefit between the two approaches, even though such programs are expensive and time-consuming. Although these studies do not rule out the role of hospitalization in the treatment of AN, they indicate that there is no systematic benefit to hospitalization when compared to outpatient treatment [13].

*Day Treatment Programs*

Day treatment programs (day hospitalization and partial hospitalization) can help individuals with eating disorders who are medically stable and do not require 24-hour administration but require more than outpatient treatment [17]. A multidisciplinary team provides 8-10 hours of care each day (including meals, therapy groups, and other activities) five days a week. There have been few published evaluations of child and adolescent day treatment programs, but they are regarded to play an important role in the continuum of care because they are observational in design [18].

*Outpatient Treatment*

For most patients with anorexia treated in an outpatient setting, it is effective and costs less than inpatient treatment. This is provided to children and adolescents who are medically stable and whose psychiatric risk is managed. A range of treatment options aims to educate young people and their parents about the physiological and psychological effects of food restriction and help them manage problems effectively.

*Family-Based Therapy*

For children and adolescents with eating disorders, FBT has emerged as the preferred first-line treatment. The therapy’s goals are to teach, empower, and support parents in specific methods to address the need for parents to work together, set specific behavior change goals, use consistent and persistent management techniques, reduce negotiating behaviors, and minimize emotional responses to demands and protesting verbal and physical behaviors. Therapists help parents employ their own abilities and parenting methods that have been blocked by AN, as well as adopt new ones if needed, to solve the problem. Treatment is broken down into three stages. The first stage teaches parents how to take action and alter their child’s weight maintenance behaviors so that their child begins to gain weight under their supervision. This stage typically lasts three months. The second stage begins after the child’s weight has been mostly regained and allows for a gradual age-appropriate reintroduction of eating and exercise control. It enables the patient to display sufficient mastery of these activities that advance to gradual independence in eating. This stage lasts around two to three months. The third stage occurs when the patient consumes an adequate amount of food for health and growth without parental assistance. This brief stage aims to reintroduce the child and family to the regular teenage development process. Typically, this stage lasts between one and two months. FBT takes between six to 12 months and between 10 to 20 sessions [19,20].

In a study, 80 patients were randomly assigned to either family therapy or supportive individual therapy in a controlled trial involving the following four subgroups: (a) individuals with early-onset, brief time (below three years); (b) individuals with initial start, extended duration (above three years); (c) individuals with tardy-onset (19 years or above) and extended duration (more than three years); (d) AN, BN subtype. Inpatient hospitalization was used to bring patients up to their target weight, and they were subsequently included in the therapy study following discharge. For the early-onset, short-duration group, family therapy had a better effect than individual treatment on weight, nutritional status, menstrual status, psychosexual functioning, and socioeconomic functioning based on interview-based outcome evaluations. The late-onset anorexia group, on the other hand, benefited more from individual therapy than family therapy for weight gain (but not in any other way). A total of 110 RCTs have been published since 1987, with a total of over 1,000 participants. The most extensive data is in support of a specific type of family therapy, FBT, which was initially manualized in 2001 and is now widely available for research, dissemination, and application. Other treatments, such as adolescent-focused therapy (AFT), cognitive behavioral therapy (CBT), and systemic family therapy, all have some empirical evidence but are not as considerable as FBT, which has the most available evidence [13,20].

In parent-focused therapy, the therapist assists the parents in renourishing the patient and decreasing weight control habits, but after the initial meeting, the therapist only meets with the parents. As part of the assessment process, the patient sees an individual nurse or physician for a few minutes to discuss their weight and any acute mental health difficulties [21].

A subsequent assessment was conducted to examine the significance of family therapy where the study evaluated (a) conjoint family therapy, where the entire family participated in treatment sessions, and (b) family counseling, where the parents received the same advice on managing the child’s ingesting issues without the child present. Additionally, the teens receive personal encouragement sessions. A total of 18 teens, all below the mandatory age, who were diagnosed with early-onset AN were randomly allocated into the two above-mentioned groups. After 32 weeks of treatment, the outcomes were analyzed. In terms of weight loss, eating habits, and self-esteem, the results of both family counseling and conjoint family therapy were the same. These findings show that seeing the whole family is not obligatory to yield excellent results when employing a family intervention strategy [13].

*Psychodynamic Individual Treatment*

This treatment is a combination of weekly individual sessions with the teenager and two collateral sessions with the parents every month which is known as Ego-Oriented Individual Therapy (EOIT). Each session focuses on the patient’s self-esteem and essential skills, in addition to interpersonal struggles related to their body, emotions, and socialization. It also assesses the correlation between these factors and eating habits, weight standards, and body perception, among others. If the patient is facing challenges in establishing their own identity separate from a severely dysfunctional family, the physician does not press the patient into gaining pounds or changing his or her point of view but will instead assist them in understanding a method for achieving optimal physical well-being, effectively managing familial dysfunction, and overcoming internal anxieties without resorting to self-imposed malnutrition. Patients’ concerns and their relationship to eating and body image are explored by the therapist to collaborate on constructive solutions that enhance the physical and emotional well-being of the patient. Following this, the therapist helps the patient achieve self-acceptance and a stronger ego, which allows for the restoration of normal eating and the achievement of sufficient body weight. During collateral sessions with parents, the practitioner assists in liberating individuals from the inclination to hold themselves responsible for their child’s eating disorder while providing accurate insights into the therapeutic, dietary habits, and mental components of the condition. As soon as parents stop paying attention to their kid’s eating problem signs, their own marital and personal concerns may be highlighted as areas for change in the later stages of intervention [13]. In a randomized controlled comparison of EOIT and Behavioral Family Systems Therapy (BFST), EOIT resulted in a slightly greater regaining of body weight than BFST but also showed comparable beneficial alteration in dietary behaviors, mental health, self-esteem, and interpersonal relationships with family members. The EOIT adolescent had caught up to the BFST adolescent in terms of BMI by one year after therapy [13].

*Cognitive Behavioral Therapy*

The therapist utilizes cognitive reformation in the treatment of AN to change inaccurate ideas and behaviors regarding the *meaning* of body mass, form, and physical appearance, which is thought to discourage diets and the anxiety associated with gaining weight [13]. Studies are scarce on CBT for AN, and none of the existing research has focused on children or adolescent cognitive development required to conduct these interventions. Adapted CBT-AN and Specialist Supportive Clinical Management (SSCM) were used in an RCT among women with severe and long-lasting AN (defined as more than seven years) with the primary goal of enhancing quality of life rather than weight gain. Both groups saw improvements in health-related quality of life and eating disorder outcomes, although CBT-AN participants showed more improvement in social adjustment, eating disorder symptoms, and willingness to change at follow-up. A retention rate of almost 87% was achieved for treatment completion, while 86% completed the follow-up examinations. This represents the highest retention rate among all therapy trials for individuals with AN. Research findings suggested patients who have extremely long-lasting AN could benefit from specialized therapy [22].

Enhanced cognitive behavioral therapy (CBT-E) has shown beneficial outcomes in adult settings for both AN and BN, and substantial improvements in weight and eating disorder pathology maintained over 60 weeks have been seen in an adolescent cohort. This treatment focuses on the cognitive processes maintaining the eating disorder to drive changes in eating behavior [23].

*Adolescent-Focused Therapy*

Studies have demonstrated that AFT is just as effective as FBT, even though the results of FBT are better at six and 12 months. For adolescents with AN, AFT is probably the second-best evidence-based treatment option. Families that are unable to commit to FBT for various reasons, such as illness, may benefit from this treatment alternative [24].

AFT consists of three phases. Phase one concludes with the therapist identifying the core issues. Adolescents in phase two of the treatment are taught how to cope with their issues without resorting to AN behaviors and obsessions that they had previously developed. At this point, the adolescent is either directly counseled by the therapist or, in certain situations, indirectly counseled by another expert. Consolidation, practice, and finalization with the therapist are the primary goals of the third phase, which focuses on coping with emotions and interpersonal interactions.

Counselors may use parent-child sessions to aid the parents in supporting their child’s growth and preventing adolescent suffering and experimentation. Hospitalization rates for medical instability were greater with AFT than with FBT because AFT did not focus on regulating eating behaviors [20,25].

*Pharmacotherapy*

Although several drugs have been explored for the treatment of AN, primarily in adults, none have been authorized by the US Food and Drug Administration (FDA). Despite their proven ineffectiveness, psychotropic medicines are prescribed to more than half of adolescents with restrictive eating disorders, mostly to manage comorbid conditions such as anxiety and depression. In such cases, it is more efficient to treat AN first, and only after there is a sensible response to treatment and the symptoms of comorbid disorder persist, the medication would be prescribed. Three small RCTs conducted among teenagers suggested that risperidone and olanzapine did not provide any significant benefit during the weight-restoration process. In an open-label RCT, quetiapine was found to be effective in improving both physical and psychological symptoms; nevertheless, the study was not sufficiently powered to draw strong conclusions. The use of selective serotonin reuptake inhibitors (SSRIs) for the treatment of AN symptoms or the prevention of relapse has also been found to be ineffective. Psychotropic drugs are prescribed in large quantities despite the absence of proof that they are useful [20,26,27].

Treatment for bulimia nervosa

*Inpatient Treatment*

BN patients can be treated primarily in the outpatient setting, with just a small percentage (approximately 5%) requiring inpatient care. The following conditions necessitate inpatient treatment: (a) dehydration, electrolyte abnormalities, and abnormal heartbeat patterns have all been linked to binge eating and purging. (b) Psychiatric decompensation or a pattern of self-harming actions, such as severe psychosis or recurrent suicidal ideations or actions related to suicide. (c) Overindulging and vomiting that has not improved with rigorous therapy in less strict settings (i.e., out-based clinic or partial hospital stay). (d) Additional significant overlapping conditions that make it difficult to treat BN (e.g., illegal recreational drug use and mental illnesses). However, there is a lack of research results that have examined the efficacy of inpatient therapy for teens and children with bulimia [13]. *Partial Hospitalization*

Such programs have been effectively created for children and adolescents with a variety of problems, and they may have a reasonable chance of assisting more severely disturbed bulimic adolescents. These programs are specifically developed to offer assistance and engage patients in various activities for seven to 10 hours per day throughout the week. This enables patients to go back to their homes in the evenings and on holidays [13]. The primary goals of bulimic partial hospitalization are (a) the cessation of excessive consumption, elimination, and other coping mechanisms; (b) dietary rehabilitation addition, if necessary, to increase weight; (c) identifying and confronting the underlying psychological reason that originated and sustains the eating disorder.

*Family-Based Therapy*

One-to-one therapy sessions for patients with bulimia, as well as additional sessions involving relatives, following a similar approach utilized for treating anorexia, may be the most clinically effective option [13]. Three high-quality RCTs for BN compared FBT to various control groups. When FBT was compared to CBT, the remission rates in the FBT group were significantly greater than that in the CBT group (39% versus 20%). When comparing the FBT group to the supportive psychotherapy group, the remission rates were considerably higher in the FBT group (39% versus 18%). However, when family therapy (with some elements consistent with FBT) was compared to guided self-help CBT, there were no considerable differences (10% versus 14%). The adolescents in this study were slightly older and had the choice to involve a “close other” rather than a parent.

FBT-BN was compared to a modified version of CBT that was designed for adolescents with BN (CBT-A) in a more recent study [28]. In this trial, 109 adolescents (aged 12 to 18) with a DSM-5 diagnosis of BN or partial BN were randomly assigned to either FBT-BN or CBT-A for 18 sessions over six months. FBT-BN was significantly superior to CBT-A in terms of binge eating abstinence and 25 purging episodes during the 28 days before treatment completion (39.4% versus 19.7%, p = 0.04). Abstinence rates for both groups improved at the six-month follow-up, but FBT-BN abstinence rates remained significantly higher (44% and 25.4%, respectively, p = 0.03). The abstinence rates did not differ statistically at the 12-month follow-up, and the study’s follow-up analysis was not powered [29].

*Cognitive Behavioral Therapy*

CBT for BN is a brief, targeted strategy that establishes connections between an individual’s thoughts, feelings, and actions, and its concepts apply to many disciplines. The hypothesis behind this method is that specific cognitive qualities, including diminished feelings of worth, erroneous views about their body, binary thinking, and idealistic tendencies, contribute to a highly unhealthy obsession with body dimensions. Obsession results in restricted eating (dieting), which increases physiological and psychological susceptibility to binge eating. Therefore, reestablishing regular eating patterns is a primary goal, and educating patients about the perpetuating nature of the restriction-binge-purge cycle is a priority from the start [13,30].

There are three stages of CBT for BN. Individuals in stage one are guided through psychoeducational principles and behavioral techniques to break the cycle of binge eating and purging while also helping them return to their normal eating patterns. During this phase of treatment, the practice of keeping track of one’s eating habits with the use of a food diary becomes deeply imprinted. In the second stage, a person is helped to identify and confront the distorted thoughts, attitudes, and values that sustain their eating disorder through cognitive restructuring and problem-solving. An investigation into the personal and external factors that are responsible for bulimic bouts is conducted, and alternate means of coping are developed. In the last part of the therapy, many measures for preventing a rebound are taken into consideration, along with progress maintenance. Although there have been no published studies examining the effectiveness of CBT in treating target groups with bulimia, empirical evidence suggests that such a strategy can be successful, provided that the children or teens have acquired the necessary intellectual skills, including the capacity to think conceptually about mindsets and opinions related to body build, as well as the ability to consider different viewpoints and be open to accepting them [13].

Cognitive behavioral therapycompared to other treatments: In RCTs for adolescent BN, a family approach was compared to individual psychotherapy. A total of 85 adolescents with BN or an eating disorder were randomly assigned to either family therapy or CBT-guided self-care. Adolescents engaged in CBT-guided self-care while being supported by a healthcare practitioner. The primary outcomes were abstinence from binge eating and vomiting after six months of treatment. Secondary outcomes included attitudinal bulimic symptoms and treatment costs. Adolescents who received CBT-guided self-care experienced significant reductions in binge eating after six months, but there were no differences in purging behavior or attitudinal symptoms at the end of therapy [29]. Additionally, three high-quality RCTs studying CBT have been reported. In one RCT, CBT was compared to psychodynamic treatment in adolescents and young adults. The trial found no difference in terms of BN remission. There were very minor differences in the CBT group in terms of a higher reduction in binge-purge frequency. Two RCTs compared CBT to FBT for BN. The findings of these two studies contradict each other, with the study by Le Grange et al. reporting significantly higher remission rates in the FBT group compared to the CBT group, whereas the study by Schmidt et al. finding no significant difference between the groups, with only a small proportion remitting in each study. Two large case studies revealed a considerable decrease in binge-purge frequency before and after treatment [31]. Furthermore, an RCT contrasted two groups one managed by adjunctive fluoxetine following CBT and the other managed by stepped care involving self-help, followed by adjunctive fluoxetine and CBT). At a one-year follow-up, remission rates were not significantly different (CBT group: 44%, stepped care: 32%); however, stepped care outweighed the CBT group in lowering compensatory behaviors and binge eating. Stepped care had higher rates of abstinence (25%) among those projected to be non-responders than CBT (4%). CBT was more expensive than stepped care. According to the findings, stepped care may be a useful strategy for patients who are anticipated to be non-responders, and supported self-help may be a valuable substitute for CBT when utilized in a stepped-care sequence [32]. Finally, by targeting eating disorder-maintaining components such as nutrient rehabilitation, feelings, and self-centered orientation, Integrative Cognitive-Affective Therapy (ICAT) for BN seeks to increase treatment effectiveness. An improved version of CBT was compared to ICAT (CBT-E). Although the therapies were not statistically different, the symptoms improved. At the four-month follow-up, the rates of binge-purge abstinence were almost 23% for CBT-E and 33% for ICAT. Moreover, there was no discernible difference in the proportion of patients within one standard deviation of the global EDE score community with a mean of less than 1.74 (CBT-E: 50%, ICAT: 55%). More research is needed on ICAT, especially regarding its long-term consequences. Antidepressant medication effectiveness was contrasted with CBT in two outpatient investigations. The results indicated that CBT combined with antidepressants or not was more successful than pharmacological therapy alone [13,33].

*Interpersonal Psychotherapy*

Interpersonal psychotherapy (IPT) concentrates on the essential interpersonal ties in an individual’s life that seem to have contributed to and sustained their BN. In the first stage, the patient is told that eating disorders are caused by interpersonal issues and that addressing such issues is essential to overcoming the condition of bulimia. After that, an in-depth evaluation of the adolescent’s interpersonal relationships is performed. The middle stage involves a collaborative effort between the therapist and patient to address the identified problem areas through the use of cognitive, behavioral, and interpretative strategies. Finally, the therapist assists the adolescent in terminating the therapeutic engagement and establishing a sense of competence to deal with future challenges. Parents and other close family members attend integrated appointments with the teenager regularly during all three stages of the treatment. Adolescents’ lives revolve around their intimate relations with peers; therefore, IPT should intuitively appeal to them. This will give IPT an advantage over this patient population. Recently, IPT has been modified for therapeutic application (i.e., IPT-BNm), with promising efficacy in a pilot case series. The IPT-BNm was shortened to only include 10 meetings (IPT-BN10). However, it needs further investigation [34].

*Pharmacotherapy*

Due to the limited number of published studies, evidence-based pharmacological treatment for children and adolescents with eating disorders, in general, and BN, in particular, is not yet possible. Furthermore, adult patients were the first to receive BN pharmaceutical treatment. Drugs such as SSRIs have been shown to be beneficial in treating adult BN; however, fluoxetine is the only FDA-approved medication for this purpose. It is possible to treat BN using fluoxetine, even though it is not FDA-approved for pediatric BN. Fluoxetine has been licensed for the treatment of obsessive-compulsive disorder and depression in children and adolescents. It has been demonstrated that topiramate, an antiepileptic, effectively reduces binge eating in individuals who do not respond to or tolerate SSRIs well. Topiramate has been linked to eating disorder symptoms in adolescents. One open trial of fluoxetine in 10 teenagers aged 12 to 18 years reported on an eight-week titrating dose of fluoxetine (maximum 60 mg daily) together with supportive psychotherapy. Weekly bingeing decreased from a mean of 4.1 to 0 episodes, and weekly purging decreased from 6.4 to 0.4 episodes per week. None of the participants reported any serious negative effects. Whether patients maintained these benefits over the long term is indefinite. In addition, no study has exclusively investigated how antidepressants are used among kids or teenagers. However, where there is proof of serious depressive disorder that is distinct from eating problems or when there has previously been a history of either a partial or non-response to strong psychological therapies, the use of antidepressants should be considered [29,33,35].

Treatment for binge eating disorder

*Psychotherapy*

The majority of BED patients are treated in outpatient settings with the support of medical and mental health specialists as well as, if necessary, a dietitian. BED suffers from a clear shortage of psychological treatment. However, there is limited evidence supporting CBT in the treatment of BED. CBT stresses on the interrelationships between an individual’s ideas, feelings, and behaviors, and its concepts can be used across disciplines. The goal is to reinstate regular eating behaviors, and one of the first steps is to educate patients on how to break the restriction-binge-purge cycle. If patients with BED eat consistently throughout the day, they can reduce the urge to binge which often occurs late in the day. Reducing the number and frequency of binges may help reduce guilt and shame, as well as the negative self-esteem that comes with them. Patients are encouraged to challenge their skewed views and change their eating habits during CBT. Furthermore, adolescents at risk of obesity and eating disorders who received IPT and health education showed reductions in BMI, loss of control eating, anxiety, and depression. The IPT group’s binge eating frequency goal was also reduced at the 12-month follow-up [25,30].

Two new meta-analyses have added to the body of evidence supporting CBT’s effectiveness in the treatment of BED. Hilbert et al. conducted an RCT evaluating CBT, revealing a substantial decrease in the frequency of BED bouts and complete remission of BED in CBT patients. There did not seem to be a therapy that was better at reducing bouts of binge eating when RCTs with active control groups were examined [11].

A total of 130 transdiagnostic patients were assigned to either CBT-E or IPT in five trials conducted in 2015. Post-treatment, psychopathology, in general, and eating disorder-specific terms, as well as BMI and the presence of binge eating/purging habits were assessed. Psychopathology levels dropped in both groups, although substantially more in patients assigned to CBT-E. Most patients attained resolution of BED bouts, abused laxatives less, or vomited less in comparison to IPT (45% versus 22%). Recent research has demonstrated the effectiveness of CBT and IPT at one and two-year follow-ups. Five-year outcomes were evaluated to extend these findings and test the stability of treatment benefits. CBT and IPT were shown to be equally beneficial in terms of a prolonged healing percentage: 52% for CBT and 77% for IPT) (without binge bouts). The treatments were the same at all times. Both IPT and CBT were effective treatments for BED [11,36]. Binge eating was reduced in 45.5% of those who received CBT + placebo, 36% of those who received CBT + fluoxetine, and just 5.9% of those who received fluoxetine, according to the long-term results of CBT. As a result, CBT greatly outperforms fluoxetine, and the addition of fluoxetine to CBT did not have any added advantage. It is necessary to conduct more research on patient characteristics that anticipate long-term responsiveness to CBT [36].

*Pharmacotherapy*

Research on the treatment of BED lags behind that of other eating disorders and has focused mostly on adult subjects, with little or no research undertaken on adolescent or pediatric patients. In the treatment of BED, antidepressant medications have a good evidence base, with benefits including a considerable reduction in binge eating and purging behaviors. The use of other antidepressants, including those from the SSRI and tricyclic antidepressant classes, has been shown to be statistically and clinically superior to placebo in reducing the frequency of binge eating and purging behaviors. Although fluoxetine is passably effective in reducing the frequency of binge eating episodes in individuals with BED, it does not help them lose weight. As a result, numerous overweight and obese BED patients are looking for other ways to manage their behaviors.

Only fluoxetine at a dose of 60 mg may be beneficial in controlling bingeing and purging episodes and total illness severity in adolescents, with the evidence limited to one study in this population [2,11]. It was also explored whether BED could benefit from the stimulant lisdexamfetamine, which is approved by the FDA to treat Attention-deficit/hyperactivity disorder. An 11-week clinical trial with 514 people with BED who were given lisdexamfetamine dimesylate at 30 mg, 50 mg, and 70 mg doses or placebo was conducted at 30 sites across the United States. For the 50 mg/day and 70 mg/day groups, statistically significant decreases in binge eating days/week occurred at week 11 compared to placebo; however, this result did not show up in the 30 mg per day group. The negative side effects aligned with the drug’s established safety record. Furthermore, the standard deviation in body weight was -1 (3.09) for placebo, -3.1 (3.64) kg for the 30 mg/day group, -4.9 (4.43) kg for the 50 mg/day group, -4.9 (3.93) kg for the 70 mg/day group, and -4.3 (4.09) kg for the combined therapy group. The FDA approved the use of 50 mg and 70 mg dosages for the treatment of BED after additional studies confirmed their effectiveness in treating binge eating episodes and related symptoms [11].

Treatment of avoidant/restrictive food intake disorder

*Inpatient Treatment*

Many individuals with ARFID achieve a state of stability as they remain underweight for so long; hence, they do not show up as emergency cases as AN. To assess whether the patient requires hospitalization, physicians usually track the patient’s weight; however, if the BMI of the patient is below three-fourths of the median BMI for their age and sex, admission may still be required. If a patient with ARFID is admitted to the hospital for medical reasons, he/she may become better on a controlled re-feeding strategy as they will gain weight along with the detection and and correction of any electrolyte abnormalities (risk of refeeding syndrome). Nevertheless, because ARFID patients struggle with the volume plus the diversity of their meals, it may become obligatory to feed them their favorite meals first until meeting the escalated volume aimed to aid weight gain. Patients with ARFID had the same electrolyte shifts as patients with AN, according to a retrospective chart review of medically hospitalized patients; however, patients with ARFID had a longer length of stay than patients with AN, which is thought to be due to increased reliance on enteral feeding and lower starting calorie goals early in the admission. Patients with ARFID are more prone than AN patients to rely on enteral feeding, according to a study of medically hospitalized patients with eating disorders. When selecting whether to employ supplements or food alone, the patient’s current intake, dietary restrictions, and motivation for therapy should all be taken into account. In acute malnourishment, a nasogastric (NG) tube is a life-saving therapeutic tool. However, it should be considered a temporary solution to assist the overall treatment objective of getting enough nutrients through oral intake. Drawing off an NG tube is usually performed under strict observation in a day therapy or inpatient setting when patients have reached their weight and are able to consume a reasonable amount of food orally [37].

*Outpatient Treatment*

If the patient’s health is not in danger, the physician should examine if intensive clinic-based eating disorder programs or day treatment referrals are required, or if outpatient psychotherapy is adequate on its own. A study on ARFID patients found that day treatment is a successful care plan for food restriction, weight improvement, and reduced restlessness. However, if clinical symptoms and signs are serious, or if food restriction persists, supplementation with certain vitamins and minerals should be considered [37,38].

*Psychological Treatment*

Psychological treatments for ARFID are becoming more widely available. In collaboration with the MGH Eds Clinical and Research Program, experts developed a CBT for ARFID (CBT-AR) that can be used to manage patients aged 10 and above who have typical ARFID manifestations, are not dependent on enteral eating, and are medically stable. Depending on the patient’s age, this organized outpatient care could be provided in a solo or family-supported style, and it requires 20-30 meetings depending on the level of malnourishment. The CBT-AR is based on the principle of food bulk rather than the diversity of food to achieve the patient’s dietary recovery (i.e., correction of deficiencies and weight gain). Patients who are below their normal weight are specifically encouraged to consume bigger portions of their favorite foods during the first phase of treatment, followed by increased dietary variety during the second and third stages. However, the efficacy of CBT-AR is presently being investigated at MGH, thus the results remain unknown. A published case study using the approach showed positive results in terms of resolving deficiency, weight gain, and minor expansion of food diversity [37].

*Pharmacotherapy*

Patients with ARFID may benefit from the use of cyproheptadine, an antihistamine plus antiserotonergic medication. According to a study conducted on children with a range of feeding issues, aged seven months to six years, patients receiving cyproheptadine experienced higher increased weight, improved feeding behaviors, and improved mealtimes compared to those who did not get the medication. Cyproheptadine, which promotes increased appetite and stomach accommodation, helped several patients [37]. Currently, there is no FDA-approved psychiatric drug for the treatment of ARFID. Mirtazapine was tested in patients with ARFID and was shown to be safe and effective. Mirtazapine (average daily dose of 25.5 mg, range 7.5-60 mg/day) was administered to 14 patients aged seven to 23 with ARFID for an average of 9.8 weeks. Although both studies had a small sample size, the results showed that mirtazapine can help patients with ARFID gain weight more quickly, with a statistically significant difference between the average change in BMI per week before mirtazapine and after mirtazapine of 0.23 BMI points per week. Low-dose olanzapine (3 mg/day) was studied in a retrospective case review of nine patients with ARFID in inpatient, partial hospitalization, or intensive outpatient programs in 2017. A small non-controlled investigation indicated that supplemental low-dose olanzapine appeared to reduce depression and anxiety symptoms and facilitate weight gain and ease of eating, concluding that utilizing olanzapine in ARFID is worth considering in future randomized placebo-controlled trials [37,39].

## Conclusions

Eating disorders are common among children and adolescents, and there are several types of eating disorders with different first-line therapies. For AN, FBT is considered the first treatment option, and AFT for adolescents is considered the second-best evidence-based approach. No evidence suggests the use of CBT for children and adolescents due to the minimum age and level of cognitive development, although hospitalization should be considered in severe cases. In BN, in the majority of patients treated in an outpatient setting, FBT and CBT were shown to be equally effective. IPT is thought to be effective in adolescents. Moreover, IPT and CBT are psychotherapies that appear to be beneficial for BED.

In ARFID psychotherapy, intensive outpatient treatment, day treatment, or hospitalization is considered depending on the severity of the condition. Regarding pharmacotherapy for eating disorders, none of the medications have been approved by the FDA. Psychotropic medication could be used only to treat comorbid conditions, including mood and anxiety disorders. Malnutrition symptoms might be misdiagnosed as anxiety, depression, or attention-deficit/hyperactivity disorder, although they are treated with careful renourishment and evidence-based psychotherapy. As the majority of studies examining the effectiveness of the therapy were conducted in Western countries, we recommend that studies be conducted in the rest of the world because individual countries may have different concepts affecting therapy choices.
